# Increase in diastolic blood pressure induced by fragrance inhalation of grapefruit essential oil is positively correlated with muscle sympathetic nerve activity

**DOI:** 10.1186/s12576-020-00733-6

**Published:** 2020-01-30

**Authors:** Eriko Kawai, Ryosuke Takeda, Akemi Ota, Emiko Morita, Daiki Imai, Yuta Suzuki, Hisayo Yokoyama, Shin-ya Ueda, Hidehiro Nakahara, Tadayoshi Miyamoto, Kazunobu Okazaki

**Affiliations:** 1 Department of Environmental Physiology for Exercise, Osaka City University Graduate School of Medicine, 3-3-138 Sugimoto Sumiyoshi, Osaka, 558-8585 Japan; 2 Research Center for Urban Health and Sports, Osaka City University, Osaka, Japan; 3 Department of Physical Education, Faculty of Education, Gifu University, Gifu, Japan; 4 Graduate School of Health Sciences, Morinomiya University of Medical Sciences, Osaka, Japan; 5 Department of Sport and Health Sciences, Faculty of Sport and Health Sciences, Osaka Sangyo University, Daito, Osaka, Japan

**Keywords:** Blood pressure, Autonomic nerve, Aromatherapy

## Abstract

Fragrance inhalation of essential oils is widely used in aromatherapy, and it is known to affect blood pressure (BP) and heart rate (HR) via autonomic control of circulation. In this study, we aimed to test the hypothesis that the changes in hemodynamics with fragrance inhalation were observed along with changes in muscle sympathetic nerve activity (MSNA). In study 1, thirteen healthy men were exposed to fragrance stimulation of grapefruit essential oil for 10 min, and BP, HR, and MSNA were continuously measured. In study 2, another nine healthy men were exposed to the same fragrance stimulation; responses in BP and HR were continuously measured, and plasma noradrenaline and cortisol concentrations were determined. We found that diastolic BP increased significantly during fragrance inhalation, while the other variables remained unchanged in both studies. Although MSNA burst frequency, burst incidence, and total activity remained unchanged during fragrance inhalation, we found a significant linear correlation between changes in diastolic BP in the last 5 min of fragrance inhalation and changes in MSNA burst frequency. The plasma cortisol concentration decreased significantly at 10 min of fragrance inhalation, though the noradrenaline concentration remained unchanged. These results suggest, for the first time, that changes in BP with fragrance inhalation of essential oil are associated with changes in MSNA even with decreased stress hormone.

## Introduction

Aromatherapy is widely used for variety of purposes [1–4]. Generally, essential oils extracted from plants or fruits are used for aromatherapy as the fundamental fragrance component. Recent studies revealed that aromatherapy using essential oils may provide clinical benefits and could be used as an alternative medical treatment for hypertension [1, 2, 5, 6], hypotension [1, 3], cognitive dysfunction [4], and physical and psychological stress and exhaustion [2, 7–11]. For example, Fernandez et al. [3] reported that the anti-hypotensive effect of exposure to 1 mL of rosemary essential oil every 8 h was maintained in hypotensive patients when compared to the pretreatment period. In another study, Goepfert et al. [1] demonstrated that the systolic and diastolic blood pressure (BP) and heart rate (HR) of patients in palliative care were decreased after exposure to lavender essential oil for 10 min when compared to a placebo trial.

As for the physiological effects of aromatherapy, the responses of cardiovascular variables, including BP and HR, to fragrance inhalation of essential oils have been investigated [1–3, 5, 6, 12–16]. In experimental animals, it has been reported that olfactory stimulation with the scent of grapefruit essential oil elevates mean BP [13, 14, 16, 17], whereas olfactory stimulation with the scent of lavender essential oil decreases mean BP [15, 16]. Similarly, in humans, Sayorwan et al. [5] demonstrated that systolic and diastolic BP and HR decreased with fragrance inhalation of lavender essential oil compared with the control condition. We also reported that mean BP and HR decreased with fragrance inhalation of sweet marjoram essential oil when compared to the control condition [12].

The change in hemodynamics with fragrance inhalation of essential oils is reportedly associated with changes in the mechanisms responsible for the autonomic control of circulation. In experimental animals, Niijima et al. [18] reported that olfactory stimulation with grapefruit essential oil evoked a slight increase in the nerve activity of the sympathetic branch innervating the white adipose tissue of the epididymis. In addition, Tanida et al. [16] demonstrated that the elevation in mean BP with olfactory stimulation with grapefruit essential oil was observed alongside increased renal sympathetic nerve activity. In humans, one previous study using power spectral analysis of BP fluctuations in normal adults reported that fragrance inhalation of essential oils such as pepper, grapefruit, estragon, or fennel oil induced a 1.7- to 2.5-fold increase in the low-frequency component of systolic BP, indicating increased sympathetic nerve activity. On the other hand, fragrance inhalation of rose oil or patchouli oil resulted in an approximate 40% decrease in the index compared to the control, indicating decreased sympathetic nerve activity [19]. Moreover, in the study, fragrance inhalation of rose oil resulted in a 30% decrease in plasma adrenaline concentration [19]. However, no previous study has provided direct evidence showing the association between changes in sympathetic nerve activity and changes in hemodynamics with fragrance inhalation of essential oils in humans.

Sympathetic nerve activity can be measured directly in humans via microneurography [20–22]. This technique allows for direct measurement of electrical activity in postganglionic sympathetic nerves using a minimally invasive, approach in which tungsten electrodes are percutaneously inserted into peripheral nerves [20, 23, 24]. Therefore, the aim of this study was to evaluate the effects of fragrance inhalation of grapefruit essential oil, which has been reported to increase sympathetic nerve activity and BP in rats [25], on changes in hemodynamics and muscle sympathetic nerve activity (MSNA) in humans (study 1). We hypothesized that fragrance inhalation of grapefruit essential oil would induce an increase in BP, and that this increase would be associated with changes in MSNA. Additionally, to elucidate whether the observed effects of fragrance inhalation of grapefruit essential oil on hemodynamics and MSNA were induced through a stress response to the fragrance inhalation, we also evaluated plasma cortisol concentrations and the participants’ subjective emotions related to the fragrance (study 2). We also determined plasma adrenocorticotropic hormone (ACTH) concentrations in addition to cortisol and catecholamine to evaluate the effects of the fragrance inhalation on the activity of the hypothalamic–pituitary–adrenal axis and the sympathetic–adrenal–medullary axis (study 3). The results of this study will provide a better understanding of the mechanism of the changes in hemodynamics with fragrance inhalation of essential oils and aid us in developing effective strategies for the use of aromatherapy in clinical settings.

## Methods

### Subjects

In study 1, thirteen healthy male volunteers participated. Their age, height and body weight were 21 ± 2.1 years, 173 ± 5.6 cm, and 69 ± 8.3 kg [means ± standard deviation (SD)], respectively. In study 2, another nine healthy male volunteers participated. Their age, height, and body weight were 21 ± 2.2 years, 173 ± 6.2 cm, and 71 ± 15 kg, respectively. In study 3, additional nine healthy male volunteers participated. Their age, height, and body weight were 23 ± 2.8 years, 172 ± 4.2 cm, and 76 ± 22 kg, respectively. Exclusion criteria for the recruitment of subjects in both studies were: those diagnosed with cardiovascular, hypertension, respiratory, metabolic, or endocrine disease; and those who smoked or were taking prescribed medication.

### Fragrance inhalation

To examine the effects of fragrance inhalation of grapefruit essential oil on MSNA, hemodynamics, respiratory variables, autonomic control of circulation, and stress hormone, all subjects in studies 1 and 2 were tested while inhaling plain air (baseline) and while inhaling the fragrance of grapefruit essential oil. In study 3, subjects were tested at baseline and while inhaling air containing no fragrance or air containing fragrance in random order to exclude any effects of the circadian rhythm on the hormonal variables. We used a quantitative and accurate technique developed in our previous study to administer the fragrance [12]. Briefly, undiluted grapefruit essential oil (Citrus paradise oil; Seikatsunoki, Tokyo, Japan) was diffused at a rate of 0.27 mL/min using an ultrasonic aroma diffuser (DOSHISHA DAM-1101, Doshisha Corporation, Osaka, Japan) into an acrylic box (60 cm × 60 cm × 60 cm) with two drain hoses. Flow air was injected at a constant speed (30 L/min) into the box via a drain hose and controlled using a gas regulator. Thus, the essential oil diffused in the box was diluted with injected air at a given concentration (0.9 × 10^−2^ mL/L). The air containing fragrance was collected into a Douglas bag (200 L) attached to the other drain hose. Subjects wore a face mask with one-way valves throughout experiment and inhaled either air containing no fragrance or air containing fragrance from the Douglas bag. We used a three-way stopcock with balloon valves to switch the lines. Exhaled gases were collected through a hose attached to another Douglas bag to avoid diffusion of the fragrance into the room.

### Protocol

In studies 1 and 2, the experiment was performed in the midmorning (study 1, *n* = 8; study 2, *n* = 6) or the afternoon (study 1, *n* = 5; study 2, *n* = 3). In study 3, all experiments were performed in the afternoon to evening. Subjects were instructed to abstain from consuming caffeinated or alcoholic beverages and to refrain from vigorous physical activity for at least 24 h before the experiment. Subjects arrived at the laboratory having fasted for at least 2 h after a light meal. The experiment was performed in quiet, environmentally controlled laboratories with an ambient temperature of ~ 28.0 °C with the subjects in a supine position. The concentration of essential oil component in the air and the duration of inhalation were determined from results of pilot studies.

### Study 1

After instrumentation, and at least 10 min after a satisfactory nerve recording site had been determined, the experiment was started. While the subjects remained resting in a supine position and breathing through the facemask, 5-min baseline inhalation and 10-min fragrance inhalation were completed in random and counter-balanced orders. The fragrance inhalation period was followed by a 10-min recovery period. MSNA, hemodynamics, and respiratory variables were continuously recorded.

### Study 2

After instrumentation, subjects were placed in the supine position and an intravenous catheter was inserted into an antecubital vein of the left arm for blood samples. At least 20 min after the insertion, the experiment was started. In the same manner as in study 1, 5-min baseline inhalation and 10-min fragrance inhalation were completed. Blood samples were taken after 5 min of baseline inhalation and after 5 and 10 min of fragrance inhalation.

### Study 3

All of the procedures before the start of test were same as in study 2. Subjects underwent the control trial and the fragrance trial in random order. After 10-min baseline, subjects inhaled air containing no fragrance (control trial) or air containing fragrance (fragrance trial) for 10 min. At least 20 min for recovery was inserted between trials. Blood samples were taken after 10 min of baseline and at 5 and 10 min after the start of inhalation.

## Measurements

### Study 1

#### Muscle sympathetic nerve activity

MSNA signals were obtained using microneurography [20, 26, 27]. Briefly, a recording electrode was placed in the left peroneal nerve fascicles at the popliteal fossa. A reference Ag–AgCl electrode was placed at the skin surface 2–3 cm apart from the recording electrode. The nerve signals were amplified (gain 100,000), band-pass filtered (0.7–3 kHz), full-wave rectified, and integrated by a capacitance integrated circuit with a time constant of 0.1 s to obtain a mean voltage neurogram using isolated amplifiers (MEG-1251, Nihon Kohden, Tokyo, Japan) and an integrator (E1-601G, Nihon Kohden, Tokyo, Japan). Criteria for adequate MSNA recording without any skin sympathetic nerve signals included (1) pulse synchrony; (2) facilitation during the hypotensive phase of the Valsalva maneuver, and suppression during the hypertensive overshoot phase after release; (3) increases in response to breath holding; and (4) insensitivity to emotional stimuli, deep breath, or gentle skin touch within the innervated area [26]. Before starting the experiment, we waited for at least 10 min after we observed stable data on HR, BP, and MSNA signals, to avoid any effect of the maneuvers of sympathetic stimulation for checking MSNA signals on the measurements.

#### Hemodynamics, respiratory variables, and subjective emotion related to the fragrance

R–R intervals (RRI) and HR were obtained from lead II of the electrocardiogram tracings (BSM-7201; Nihon Kohden Co., Tokyo, Japan) and beat-by-beat blood pressure (BP) was recorded noninvasively using finger photoplethysmography (Finometer MIDI; Finapres Medical System, Amsterdam, the Netherlands). Mean BP was calculated as [systolic BP (SBP) − diastolic BP (DBP)]/3+DBP. Respiratory variables were determined from oxygen and carbon dioxide fractions in the expired gas and the ventilatory volume (AE-310 s, Minato, Osaka, Japan).

Immediately after each experimental trial, the subjects were asked to rate their valence (0, unpleasant; to 9, pleasant) and arousal (0, relaxing; to 9, stimulating) [28] to allow us to evaluate their subjective emotion related to the fragrance using a 10-point scale.

### Study 2

#### Hemodynamics, respiratory variables, and subjective emotion related to the fragrance

HR was obtained as in study 1. Beat-by-beat BP was recorded noninvasively by tonometry (BP-608 Evolution II, Omron-Colin, Tokyo, Japan). Respiratory variables were determined from oxygen and carbon dioxide fractions in the expired gas and the ventilatory volume (ARCO2000-MET, Arcosystem, Chiba, Japan). Subjective emotions related to the fragrance were obtained as in study 1.

#### Blood constituents

Blood samples were transferred to a vacuum blood-sample tube containing 1.5 mg/mL EDTA-2Na and centrifuged at 5 °C for 15 min. The separated plasma sample was stored at −80 °C operating point until used to measure the plasma concentrations of cortisol (chemiluminescent immunoassay, LSI, Tokyo, Japan) and noradrenaline (high-performance liquid chromatography, LSI).

### Study 3

#### Blood constituents

The procedures for blood constituents were same as in study 2. The plasma concentrations of cortisol and ACTH (chemiluminescent immunoassay, SRL, Tokyo, Japan), adrenaline and noradrenaline (high-performance liquid chromatography, SRL) were determined.

### Data analysis

Data were stored on a computer (500 Hz sampling rate) using a computer-based data acquisition and analysis system (Powerlab 16SP and LabChart 7; ADInstruments, Sydney, Australia). Beat-by-beat HR, RRI, SBP, and DBP were extracted from the obtained data, and MSNA bursts were identified from the integrated neurogram using a MATLAB program (R2018b, The MathWorks, Natick, MA) with a 3:1 signal-to-noise ratio threshold within a 0.5 s search window and an expected burst reflex latency of 1.2 s from the preceding R waves [29, 30]. MSNA bursts were confirmed by an experienced microneurographer. Quantitative indices of MSNA were the number of bursts per minute (burst frequency, bursts/min), the number of bursts per 100 heart beats (burst incidence, bursts/100 beats), and total activity (total MSNA, units).

Sympathetic and cardiovagal baroreflex sensitivity (BRS) were calculated using data obtained during the last 2 min of baseline and fragrance inhalation. Sympathetic BRS was assessed using the slope of the linear correlation between total MSNA or MSNA burst incidence and DBP calculated over a 3-mmHg bin during spontaneous breathing after statistical weighting [31]. Cardiovagal BRS was also assessed using the slope of the linear correlation between changes in RRI or HR and changes in SBP [32]. The SD of RRI, HR, SBP, and DBP were also calculated [29, 33].

### Statistical analysis

All data were analyzed using a statistical software (SigmaPlot 14.0, Systat Software, Inc., San Jose, USA). In study 1, the minute average was calculated for each variable. Fragrance inhalation was separated into two phases: the first and last 5 min. The two phases were compared with the 5-min baseline value. Two-way analysis of variance (ANOVA) with repeated-measures (trial, BL vs. inhalation; time) was used to test the effects of fragrance inhalation on each variable. Subsequent post hoc tests to determine significant differences among various pairwise comparisons were performed using Fisher’s least significant difference test. The changes in DBP (ΔDBP) and MSNA burst frequency (Δburst frequency) with fragrance inhalation were calculated by subtracting the average value of 5-min baseline from the average value of the last 5-min of inhalation. Pearson’s product-moment correlation coefficient was used to evaluate the relationships between ΔDBP and Δburst frequency or the delta values and the baseline values. Unpaired t-test was used to determine significant difference in ΔDBP between the groups of subjects in the midmorning and in the afternoon. In study 2, the 5-min average was calculated for hemodynamics and respiratory variables. One-way ANOVA with repeated-measures (BL vs. inhalation) was used to test the effects of fragrance inhalation on each variable. In study 3, two-way ANOVA with repeated-measures (trial, BL vs. inhalation; time) was used to test the effects of fragrance inhalation on each variable. Subsequent post hoc tests to determine significant differences among various pairwise comparisons were performed using Duncan’s test. Values are expressed as the means ± SDs except as noted. *P* < 0.05 was considered statistically significant.

## Results

### Study 1

**Fig. 1:**
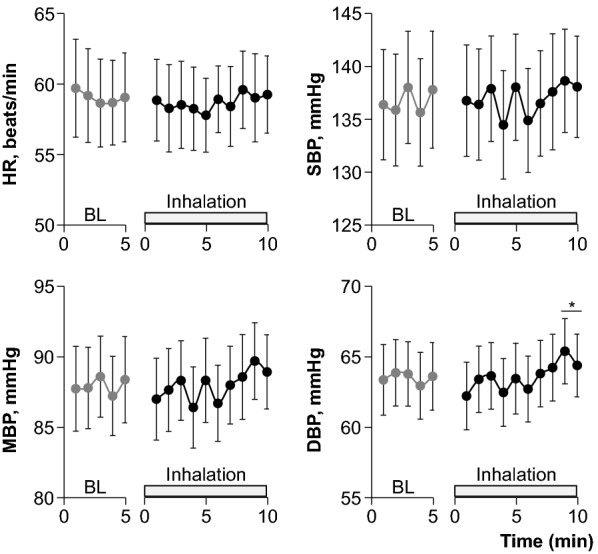
Hemodynamic responses to fragrance inhalation of grapefruit essential oil in study 1. *BL* baseline, *HR* heart rate, *SBP* systolic blood pressure, *MBP* mean blood pressure, *DBP* diastolic blood pressure. Values are expressed as the means ± standard errors. *P < 0.05 vs. BL

Figure 1 shows the hemodynamic responses to fragrance inhalation of grapefruit essential oil. HR remained unchanged, whereas BPs tended to increase during fragrance inhalation. We found a significant interaction effect (trial × time, *P* = 0.035) on DBP, which showed a significant increase at 9 to 10 min of fragrance inhalation when compared to the baseline. As shown in Table 1, respiratory variables remained unchanged during fragrance inhalation.

**Table 1:** Respiratory variables during baseline and fragrance inhalation of grapefruit essential oil in study 1

	BL	Inh-5 min	Inh-10 min
*V*_E_, L/min	8.4 ± 2.9	8.5 ± 2.8	8.1 ± 3.3
RR, breaths/min	16.2 ± 5.2	16.2 ± 4.9	16.1 ± 4.8
*V*_O2_, mL/min	236 ± 91	239 ± 93	230 ± 96
*V*_CO2_, mL/min	196 ± 80	207 ± 89	194 ± 89
RER	0.83 ± 0.08	0.86 ± 0.11	0.84 ± 0.12

Values are means ± SD. Average data for 5 min of baseline (BL) and for the first (Inh-5 min) and last half (Inh-10 min) of fragrance inhalation are shown

* V*_*E*_, minute ventilation; *RR,* respiratory rate, *V*_*O2*_, oxygen consumption rate; *V*_*CO2*_, carbon dioxide production rate; *RER,* respiratory exchange ratio

**Fig. 2:**
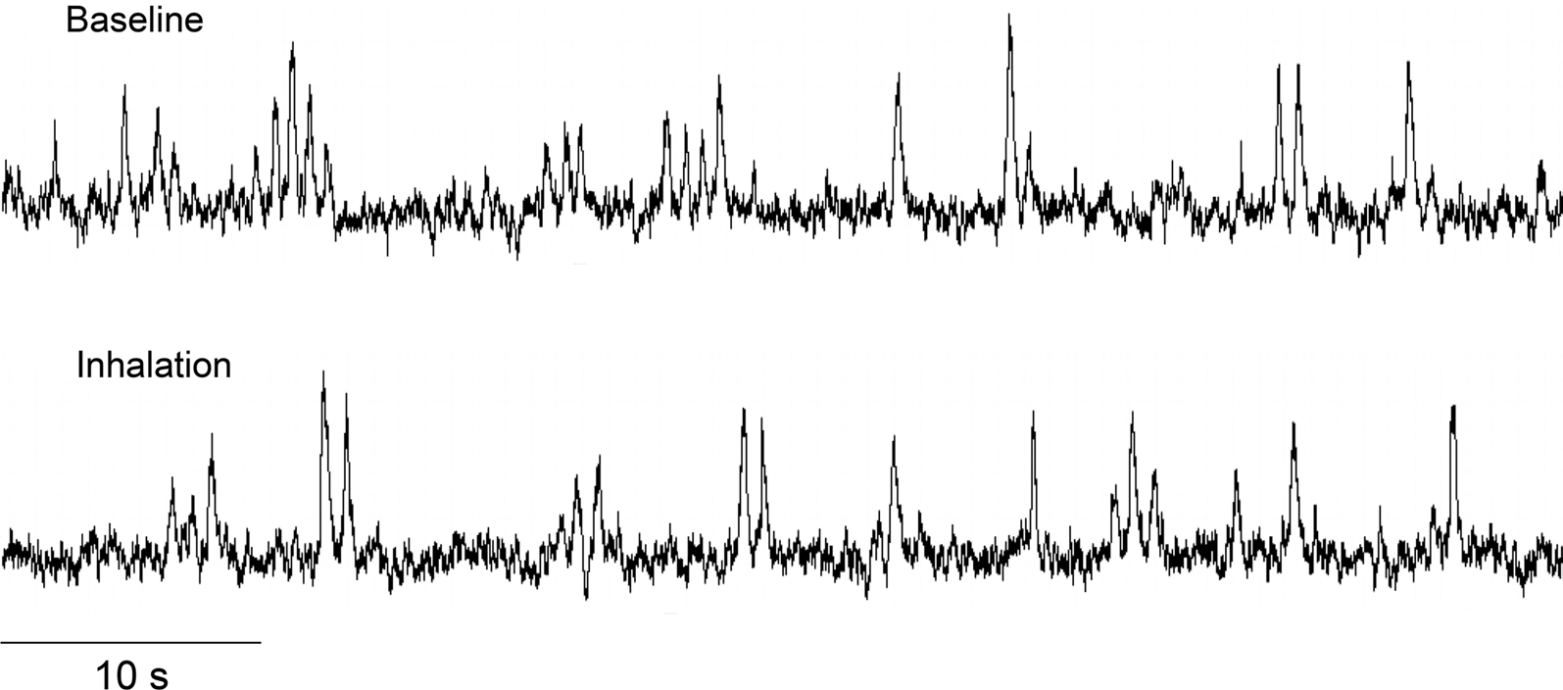
Original recordings of the integrated muscle sympathetic nerve activity from one representative subject in study 1

**Fig. 3:**
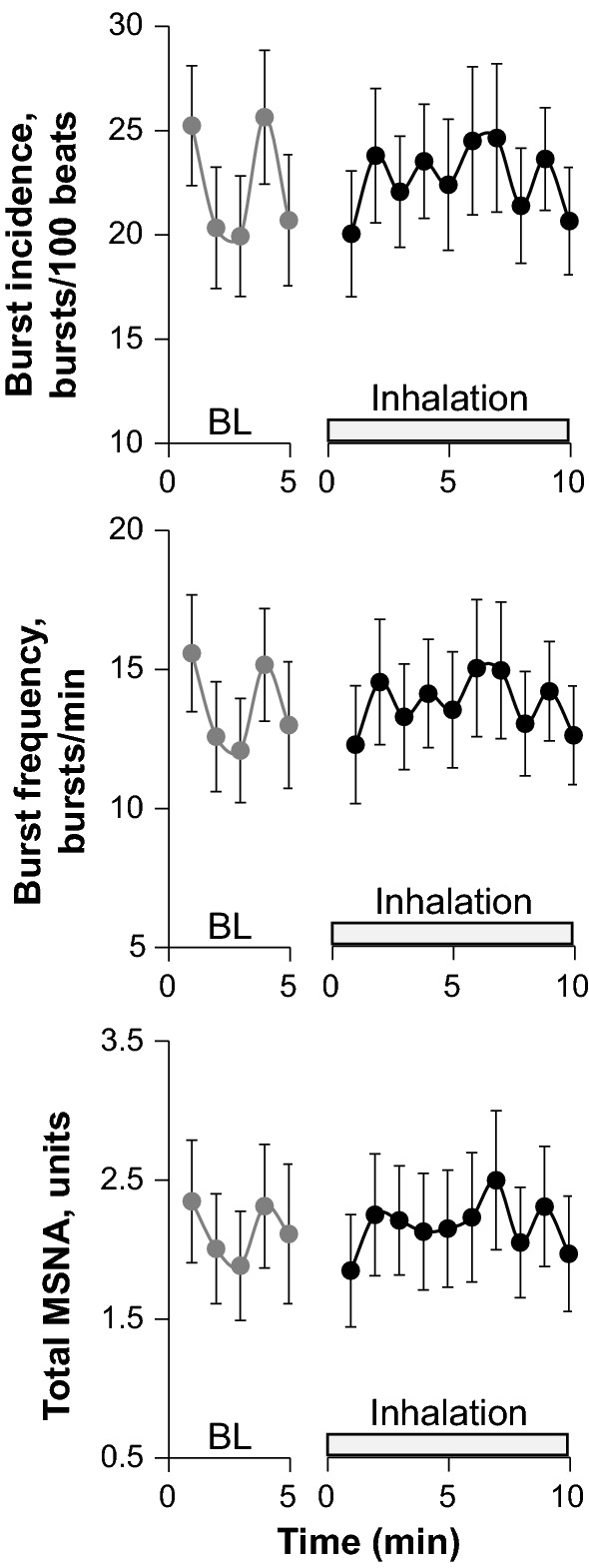
Responses of muscle sympathetic nerve activity (MSNA) variables to fragrance inhalation of grapefruit essential oil in study 1. *BL* baseline. Values are expressed as the means ± standard errors

**Fig. 4:**
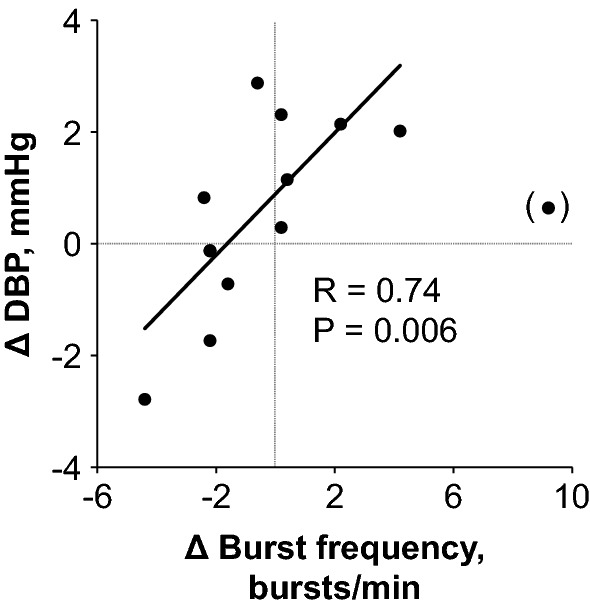
Relationship between changes in diastolic blood pressure (ΔDBP) with fragrance inhalation of grapefruit essential oil from baseline and those in burst frequency of MSNA (Δ burst frequency). Values are calculated as [5 min average for last half of fragrance inhalation] − [5 min average for baseline] for each individual. Data from one subject (shown in parentheses) was excluded from the analysis as an outlier in the Grubbs test

Figure 2 shows original recordings of the integrated MSNA from one representative subject during baseline and fragrance inhalation. Responses of MSNA variables to fragrance inhalation of grapefruit essential oil are shown in Fig. 3. We did not find any significant change in burst incidence, burst frequency, or total MSNA with fragrance inhalation. Nonetheless, as shown in Fig. 4, we found a significant linear correlation (*R* = 0.74, *P* = 0.006) between Δburst frequency and ΔDBP, indicating that the changes in DBP with fragrance inhalation were associated with changes in MSNA. Importantly, a partial correlation coefficient was significant even when MSNA burst frequency (*R* = 0.82, *P* < 0.001) or DBP (*R* = 0.72, *P* = 0.006) at baseline was included as a variable. Furthermore, there were no significant correlations between MSNA burst frequency at baseline and Δburst frequency (*R* = −0.14, *P* = 0.65) or DBP at baseline and ΔDBP (*R* = 0.24, *P* = 0.42). Intriguingly, ΔDBP showed a significant negative linear correlation with MSNA burst frequency at baseline (*R* = −0.58, *P* = 0.037), while there were no significant correlations between DBP and MSNA variables at baseline (*P* > 0.50). ΔDBP was not significantly different between the groups of subjects in the midmorning and in the afternoon (*P* = 0.84).

Sympathetic and cardiovagal BRS are summarized in Table 2. Sympathetic BRS calculated between total MSNA or MSNA burst incidence and DBP and cardiovagal BRS calculated between RRI or HR and SBP remained unchanged with fragrance inhalation. We confirmed that the relationships between total MSNA and DBP (*R*^2^ value; BL, 0.52 ± 0.35; inhalation, 0.57 ± 0.33), MSNA burst incidence and DBP (*R*^2^ value; BL, 0.54 ± 0.29; inhalation, 0.61 ± 0.32), RRI and SBP (*R*^2^ value; BL, 0.41 ± 0.15; inhalation, 0.41 ± 0.15), and HR and SBP (*R*^2^ value; BL, 0.41 ± 0.16; inhalation, 0.42 ± 0.15) were all significant in each subject. There were no significant effects of inhalation on the SDs of RRI (BL, 71.5 ± 33.4 ms; inhalation, 66.1 ± 34.7 ms; *P* = 0.512), HR (BL, 4.2 ± 2.1 bpm; inhalation, 4.1 ± 2.0 bpm; P = 0.908), SBP (BL, 7.8 ± 3.9 mmHg; inhalation, 6.4 ± 1.9 mmHg; *P* = 0.126), or DBP (BL, 4.1 ± 1.5 mmHg; inhalation, 3.9 ± 1.0 mmHg; *P* = 0.599).

**Table 2:** Sympathetic and cardiovagal baroreflex sensitivity during baseline and fragrance inhalation of grapefruit essential oil in study 1

	BL	Inhalation
Sympathetic baroreflex sensitivity		
DBP-total MSNA, units/mmHg	− 0.66 ± 0.73	− 0.86 ± 0.82
DBP-burst incidence, bursts·100 beats^− 1^·mmHg^− 1^	− 1.3 ± 1.8	− 2.0 ± 1.9
Cardiovagal baroreflex sensitivity		
SBP-RRI, ms/mmHg	10.8 ± 5.6	11.9 ± 9.6
SBP-HR, bpm/mmHg	− 0.60 ± 0.22	− 0.66 ± 0.32

Values are means ± SD. *DBP* diastolic blood pressure, *MSNA* muscle sympathetic nerve activity, *SBP* systolic blood pressure, *RRI* R–R interval, *HR* heart rate. *BL* baseline; Inhalation, fragrance inhalation of grapefruit essential oil

**Fig. 5:**
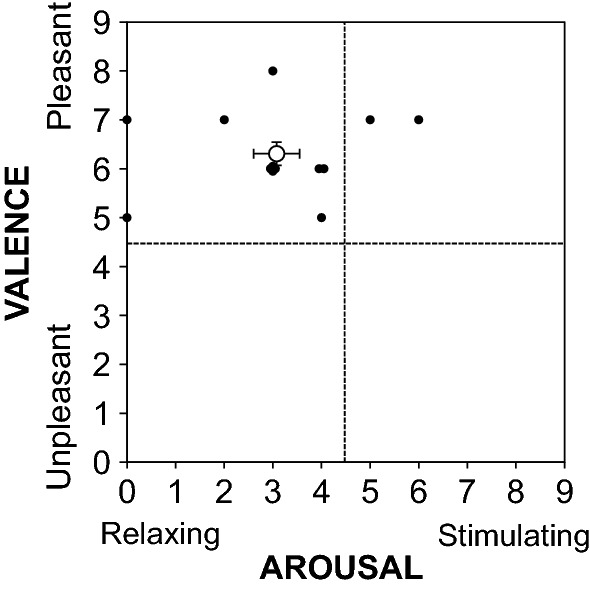
Bi-dimensional representation of the arousal and valence ratings of fragrance inhalation of grapefruit essential oil in study 1. The open circle indicates the mean ± standard errors, and the closed circles indicate the individual value for each subject

Figure 5 shows a bi-dimensional representation of the arousal and valence ratings of fragrance inhalation of grapefruit essential oil in study 1. The evaluation of subjective emotion related to the fragrance of grapefruit essential oil revealed a majority of ratings on the pleasant (valence; 6.3 ± 0.9) and relaxing parts of the scale (arousal; 3.1 ± 1.7). Similar results were obtained in study 2 (valence; 6.7 ± 1.9, arousal; 4.1 ± 2.6).

### Study 2

**Fig. 6:**
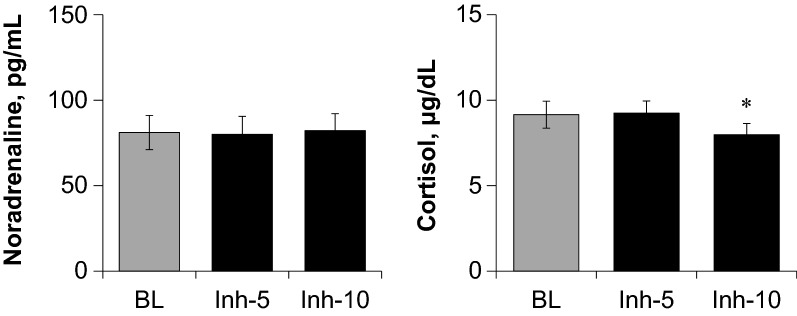
Responses of plasma concentrations of noradrenaline and cortisol to fragrance inhalation of grapefruit essential oil in study 2. Values are expressed as the means ± standard errors. BL, baseline; Inh-5 and Inh-10, at 5 and 10 min of fragrance inhalation, respectively. *P < 0.05 vs. BL

Similar to study 1 and as shown in Table 3, HR remained unchanged and DBP increased significantly during the last 5 min of fragrance inhalation when compared to baseline, respiratory variables remained unchanged during fragrance inhalation. The plasma noradrenaline level remained unchanged, whereas the plasma cortisol level decreased significantly during the last 5 min of fragrance inhalation when compared to baseline (Fig. 6).

**Table 3:** Hemodynamics and respiratory variables during baseline and fragrance inhalation of grapefruit essential oil in study 2

	BL	Inh-5 min	Inh-10 min
HR, beats/min	54.3 ± 7.4	53.8 ± 6.8	53.7 ± 7.0
SBP, mmHg	124 ± 15	126 ± 15	128 ± 3
MBP, mmHg	87 ± 13	89 ± 13	90 ± 12
DBP, mmHg	68 ± 12	70 ± 13	71 ± 12*
V_E_, L/min	8.5 ± 4.9	8.3 ± 4.6	8.3 ± 4.8
RR, breaths/min	14.2 ± 6.4	12.7 ± 6.7	13.4 ± 4.2
V_O2_, mL/min	285 ± 176	302 ± 175	290 ± 176
V_CO2_, mL/min	220 ± 136	227 ± 131	227 ± 139
RER	0.78 ± 0.06	0.76 ± 0.06	0.78 ± 0.06

Values are means ± SD. Average data for 5 min of baseline (BL) and for the first (Inh-5 min) and last half (Inh-10 min) of fragrance inhalation are shown. *P < 0.05 vs. BL

*HR *heart rate, *SBP* systolic blood pressure, *MBP* mean blood pressure, *DBP* diastolic blood pressure, *V*_E_ minute ventilation, *RR* respiratory rate, *V*_*O2*_ oxygen consumption rate,* V*_*CO2*_ carbon dioxide production rate, *RER* respiratory exchange ratio

### Study 3

**Fig. 7:**
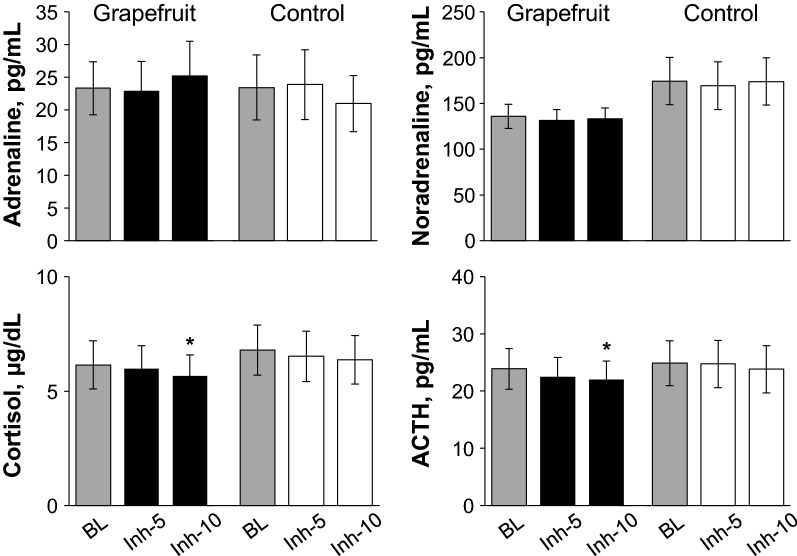
Responses of plasma concentrations of cortisol, adrenocorticotropic hormone (ACTH), adrenaline, and noradrenaline during the control trial and the grapefruit fragrance trial in study 3. Values are expressed as the means ± standard errors. *BL* baseline; Inh-5 and Inh-10, at 5 and 10 min of fragrance inhalation, respectively. *P < 0.05 vs. BL

As shown in Fig. 7, there is no significant effect of trial on the plasm adrenaline and noradrenaline levels, and those remained unchanged during fragrance inhalation in both trials. Importantly, the plasma cortisol and ACTH levels decreased significantly at 10 min of fragrance inhalation when compared to baseline in the fragrance trial while those remained unchanged in the control trial, although there are no significant effect of trial on these variables.

## Discussion

The main findings of this study are as follows: (1) DBP increased during fragrance inhalation of grapefruit essential oil while other variables remained unchanged; (2) there was a significant linear correlation between changes in DBP with fragrance inhalation and changes in MSNA burst frequency; and (3) the plasma cortisol concentration decreased with fragrance inhalation. These results suggest, for the first time, that changes in BP with fragrance inhalation of an essential oil are associated with changes in MSNA even with decreased stress hormone.

We showed that fragrance inhalation of grapefruit essential oil induced a significant increase in DBP without an increase in HR while subjects rested in the supine position. Importantly, we successfully reproduced the observations in two different studies with the same protocol (Fig. 1 and Table 3) by using a quantitative and accurate technique to apply a given fragrance [12]. Our results extended to humans previous observations on experimental animals that olfactory stimulation with the fragrance of grapefruit essential oil elevated mean BP without an increase in HR [13, 14, 16, 17].

Most importantly, for the purpose of the present study, we found that changes in DBP with fragrance inhalation were correlated with changes in MSNA burst frequency (Fig. 4). We confirmed that the changes in DBP and MSNA burst frequency with fragrance inhalation were not associated merely with baseline values. Previous studies have reported that olfactory stimulation in rats with the fragrance of grapefruit oil increased renal sympathetic nerve activity [16] or nerve activity of the sympathetic branch innervating the white and brown adipose tissues [13] and suppressed gastric vagal nerve activity [13, 18]. The present study supported these observations and suggest that, in humans, fragrance inhalation of grapefruit essential oil increased BP through the mechanisms of change in MSNA. We supposed that the changes in BP were not large enough to observe a simultaneous increase in sympathetic nerve activity, which may explain why we failed to find significant increases in MSNA variables and plasma noradrenaline concentration with fragrance inhalation in the present study. Niijima et al. [18] showed that, in rats, an olfactory stimulation with grapefruit oil (diluted 1000 times) for 10 min evoked a significant increase in sympathetic nerve activity, but not with a thinner solution (1/10,000 in concentration). It is expected that MSNA variables and plasma noradrenaline concentration may increase with a greater increase in BP if we were to use a higher/optimal concentration of grapefruit essential oil.

As for the mechanisms involved in the increase in BP and sympathetic nerve activity with fragrance inhalation of grapefruit essential oil, previous studies on rats have suggested that olfactory stimulation with the fragrance of grapefruit oil affect autonomic neurotransmission to induce an increase in BP through central mechanisms [14, 16, 34, 35]. Tanida et al. reported that olfactory stimulation with the fragrance of grapefruit [16, 34] oil or its active component limonene [14] similarly induced an elevation in renal sympathetic nerve activity and BP and suppressed gastric vagal nerve activity; moreover, intracranial injection of diphenhydramine, a histamine H1-receptor antagonist, or bilateral electrolytic lesions of the hypothalamic suprachiasmatic nucleus (SCN) completely eliminated the autonomic and cardiovascular response to grapefruit [35] and limonene [14]. Intriguingly, the elevation in renal sympathetic nerve activity and BP with olfactory stimulation with the fragrance of grapefruit oil observed in wild-type mice was not observed in clock mutant mice [16] or Cry1 and Cry2 double knockout [Cry(−/−)] mice [34]. Indeed, it has been reported that limonene is observed within brain following inhalation in mouse [36] and that the intranasal delivery of the molecule to central nerves system via axonal or extracellular transport of the olfactory nerve takes at least 5–10 min after administration [37, 38]. Therefore, it is conceivable that the increase in DBP with the changes in MSNA induced by fragrance inhalation of grapefruit essential oil observed in the present study was provoked via the SCN activated by limonene through a pathway in the olfactory system and that the central histaminergic nervous system and the molecular clock mechanism in the SCN are involved in mediating these responses. In contrast, inhalation of odor molecule activates cell of the olfactory epithelium in the nasal cavity and thus induces olfactory perception and its effect through the olfactory nervous system [39]. However, considering that the onset of olfactory perception is very rapid and acclimatization soon take place [40, 41], this mechanism would not be involved, because, in the present study, the increase in DBP required 9–10 min after the onset of inhalation. Coupled with the observations that sympathetic and cardiovagal BRS remained unchanged (Table 2), the fragrance inhalation of grapefruit essential oil seems to activate the SCN to increase the operating point of BP regulation [42].

There are substantial individual variations in the responses of DBP and MSNA to fragrance inhalation of grapefruit essential oil. Intriguingly, the changes in DBP with fragrance inhalation were negatively correlated with MSNA burst frequency at baseline, indicating that the resting level of MSNA is a determinant of the individual variations, which may be associated with the effectiveness of aromatherapy. It is inferable that the response of the SCN to fragrance inhalation of grapefruit essential oil is associated with the baseline level of sympathetic tone. Hemodynamics and sympathetic regulation of BP at baseline and in response to pressor stimulus [43], as well as vasoconstriction in response to norepinephrine and β-adrenergic vasodilation, are known to be influenced by gender [44]. Our results are limited to men; therefore, inclusion of women may alter our findings. Further studies including women are required to elucidate the background mechanisms involved in the individual variations in cardiovascular and sympathetic responses to fragrance inhalation of grapefruit essential oil.

As far as we know, no previous study has reported the direct effects of grapefruit essential oil on blood vessels, though it has been known that olfactory receptor expression is observed in the aorta, renal, and iliac arteries and in the smooth muscle cells of small blood vessels in a variety of tissues including the heart, diaphragm, skeletal muscle, and skin [45]. It has been reported that intravenous injections of essential oil of Aniba rosaeodora induce a hypotensive response and that this response remained unchanged by pretreatment via bivagotomy [46]. Although we may not be able to exclude the effects of grapefruit essential oil on BP via local vasoconstrictive mechanisms; the involvement of these mechanisms would be low, as we observed the significant correlation between the changes in DBP and MSNA burst frequency with fragrance inhalation.

We observed that plasma cortisol level decreased with ACTH level during fragrance inhalation of grapefruit essential oil (Figs. 6 and 7), and determined the subjective emotions related to the fragrance to be pleasant and relaxing (Fig. 5). In contrast, plasma adrenaline and noradrenaline levels remained unchanged during fragrance inhalation of grapefruit essential oil (Figs. 6 and 7). These observations indicate that the increase in DBP and the changes in MSNA induced by fragrance inhalation of grapefruit essential oil were not induced through a stress response which enhances both of the sympathetic–adrenal–medullary axis and the hypothalamic–pituitary–adrenal axis [47]. Recently, Takagi et al. [48] reported that fragrance inhalation of grapefruit essential oil recovers the reduction in the salivary level of secretory immunoglobulin A by mental stress in humans, indicating that the inhalation of grapefruit essential oil induced stress free actions. In addition, previous studies suggested that fragrance administration of oils though other than grapefruit essential oil attenuated an increase in salivary cortisol concentration to mental stress in humans [49, 50] or plasma ACTH level to physical stress in rats, while decreased the stress-induced activity of prefrontal cortex which regulates the activity of the hypothalamic–pituitary–adrenal axis in humans [51]. Indeed, we confirmed that plasma ACTH level decreased with cortisol level with the grapefruit fragrance inhalation (Fig. 7). These observations would indicate that the inhalation of grapefruit essential oil has a mechanism to decrease the activity of the hypothalamic–pituitary–adrenal axis. Importantly, cortisol has effects in the control of vascular smooth muscle tone by its permissive effects in potentiating vasoactive responses to catecholamines [52] and therefore might be associated with the cardiovascular changes with the fragrance inhalation. However, based on our observations that DBP increased while plasma cortisol level decreased with the grapefruit fragrance inhalation, we assumed that the increased DBP was not associated with the changes in cortisol but associated with the changes in MSNA.

### Limitations

We did not determine the effects of fragrance inhalation of grapefruit essential oil on renin–angiotensin system and vasopressin levels which would be a possible mechanism of the observed changes in DBP via vasoconstriction. As far as we know, there is no previous study reporting the effects of fragrance inhalation of grapefruit essential oil on these mechanisms. Further studies are required to assess the involvement of these mechanisms on the cardiovascular changes after the grapefruit fragrance inhalation.

## Conclusion

In conclusion, the fragrance inhalation of grapefruit essential oil induced an increase in DBP in healthy men. The changes in DBP with fragrance inhalation were correlated with changes in MSNA, even with decreased plasma cortisol concentrations. These results suggest, for the first time in humans, that the changes in BP with fragrance inhalation of an essential oil are associated with changes in MSNA. The activation of sympathetic nerve activity with fragrance inhalation without an increase in stress hormone may be one of the mechanisms involved in the positive effects and refreshment of aromatherapy in humans.

## Data Availability

Not applicable.
